# Microwave ablation *vs*. surgical resection for treatment naïve hepatocellular carcinoma within the Milan criteria: a follow-up of at least 5 years

**DOI:** 10.20892/j.issn.2095-3941.2020.0625

**Published:** 2021-09-30

**Authors:** Jianping Dou, Zhigang Cheng, Zhiyu Han, Fangyi Liu, Zhen Wang, Xiaoling Yu, Jie Yu, Ping Liang

**Affiliations:** 1 Department of Interventional Ultrasound, Chinese PLA General Hospital, Beijing 100853, China

**Keywords:** Microwave, surgery, hepatocellular carcinoma

## Abstract

**Objective:**

Thermal ablation poses challenges in the surgical resection (SR) of small hepatocellular carcinoma (HCC), and its therapeutic outcomes for larger lesions remain debated.

**Methods:**

This retrospective study evaluated 729 patients with HCC meeting the Milan criteria, who were treated with curative SR or microwave ablation (MWA) between 2008 and 2014. Overall survival (OS), cancer-specific survival (CSS), disease-free survival (DFS), and local tumor progression (LTP) were compared after propensity score matching (PSM). Co-variates associated with OS, CSS, LTP, and DFS were identified. The risk of death and tumor progression were compared.

**Results:**

During the median follow-up of 78.6 months, 253 patients were included in each group after PSM. For tumors ≤ 3.0 cm and 3.1–4.0 cm, MWA achieved comparable results in terms of OS, CSS, DFS, and LTP. For tumors 4.1–5.0 cm, MWA had lower OS, CSS, and DFS rates (all *P* < 0.05) than SR. Higher LTP rates were observed in the MWA group for tumors 4.1–5.0 cm, although the difference was not significant (*P* = 0.18). Complication rates (*P* = 0.41) were similar, but MWA led to less estimated blood loss (*P* < 0.01) and shorter postoperative hospitalization times (*P* < 0.01).

**Conclusions:**

MWA achieved comparable long-term oncologic outcomes with SR for ≤ 4 cm HCC, with lower complication rates and faster recovery.

## Introduction

Surgical resection (SR) and radiofrequency ablation (RFA) are 2 potentially curative treatments recommended by multiple international guidelines for patients with ≤ 3 cm hepatocellular carcinoma (HCC)^1,2^. For HCC larger than 3 cm, SR is currently recommended as a frontline treatment, because the extent of tumor necrosis after RFA appears to be negatively correlated with tumor size and decreases significantly with increasing tumor size^1,3–6^. RFA has not been found to be sufficient for > 3 cm HCC treatment.

Efforts have been made to enlarge the thermal field for RFA, to break the 3 cm tumor barrier in thermal ablation. Although multiple electrode placement^7–9^ and RFA plus transcatheter arterial chemoembolization (TACE)^10,11^ have been applied, local tumor progression (LTP) remains a challenge for large HCC. No-touch multibipolar RFA, a new technique of RFA for complete tumor necrosis, provides higher intensity energy than traditional RFA, although it remains associated with greater recurrence rates than those observed with SR in tumors larger than 3 cm^3,12^.

Microwave ablation (MWA) has become an increasingly used local ablation modality. Its theoretical benefits include higher intra-tumoral temperatures and a larger ablation zone than that of RFA; therefore, MWA has the potential to be used for ablation of larger tumors^13–15^. Our previous study has demonstrated that MWA shows equivalent metastasis and recurrence rates to those of SR for HCC ≤ 5.0 cm over a mean 2-year follow-up^16^. Although our short-term therapeutic responses were encouraging, the role of MWA in early-stage HCC ≥ 3.0 cm remains a topic of controversy, because other studies have reached different conclusions^17,18^. Long-term data are lacking on MWA in larger patient cohorts, addressing technique efficacy and the oncological outcomes of MWA and SR in HCC meeting the Milan criteria.

Therefore, the aim of this study was to present our 12 years’ experience, to investigate SR or MWA for patients with HCC ≤ 5 cm in terms of post-procedure and oncological outcomes with a follow-up of at least 5 years, and to provide clues for treatment selection for those patients.

## Patients and methods

### Patient selection

The study population included adult patients who received SR or MWA as a first-line treatment for HCC between January 2008 and December 2014 at a single tertiary center. Patients meeting the following criteria were included: (1) a single tumor smaller than 5.0 cm or a maximum of 3 tumors smaller than 3.0 cm; (2) Child-Pugh class A or B classification; (3) no evidence of vein or bile duct tumor embolism, and no extrahepatic metastasis at the time of diagnosis; (4) an Eastern Cooperative Oncology Group performance status of 0–1, and (5) no prior anticancer treatment. The diagnosis of HCC was confirmed with pathological results for patients who received SR. The diagnosis of HCC for patients who received MWA was confirmed either by biopsy during the MWA procedure or according to the criteria of the Practice Guidelines Committee, American Association for the Study of Liver Diseases^19^.

The institutional review board of our hospital approved this study. Written informed consent was obtained from all patients before MWA and SR treatments according to clinical protocols. Patients were categorized into SR or MWA groups according to the treatment allocation. Standardized terminology and reporting criteria for SR and MWA were used in this study^20,21^.

### MWA procedure

All MWA procedures were performed percutaneously under general anesthesia and real-time ultrasound (US) guidance. All patients underwent US, contrast enhanced ultrasound (CEUS), and contrast enhanced magnetic resonance imaging (CE-MRI)/computed tomography (CT) before MWA to access the tumor number and location, and the feasibility of US guided percutaneous MWA. A cooled-shaft MW system (KY-2000, Kangyou Medical, China) with a 15-gauge cooled-shaft antenna was used in the MWA procedure. The techniques and strategy for MWA were as described in our previous studies^22^. The ablation therapy included an ablative margin of at least 5 mm of healthy tissue surrounding the tumor. For tumors near critical structures, conformal ablation or an ablation margin less than 5 mm was achieved with the assistance of artificial pleural effusion, ascites, or temperature monitoring. If a tumor was found to be residual within 3 days after MWA, an additional session was performed to achieve complete ablation. If incomplete ablation persisted after an additional session, the case was defined as a technical failure and excluded from the present study.

### Surgical resection procedure

Surgical procedures included laparoscopic liver resection and open liver resection. Non-anatomic resection was defined as removal of the entire tumor regardless of anatomy of segment, section, or lobe. The definition of anatomic resection was the complete removal of Couinaud’s segment. In non-anatomic resection, a tumor-free margin of 5–10 mm from the tumor was achieved unless the tumor was in high-risk locations (for example, adjacent to large vessels or the diaphragm^23–25^). The choice of non-anatomical or anatomical resection was based on the tumor size, location, underlying liver disease and severity of the patient status. All surgeries were performed with standard hepatectomy techniques^26^. Intraoperative US was performed to identify tumor locations and their margins to determine the optimal dissection plane. An ultrasonic surgical aspirator was used to perform the parenchymal dissection. The liver pedicle was intermittently clamped in cycles of 10 min clamping and 5 min reperfusion when necessary.

### Patient follow-up

Therapeutic outcomes for patients in the MWA group were assessed with contrast-enhanced imaging (CE-MRI/CT or CEUS) within 3 days after the ablation to assess the technical success of ablation. Patients in the SR group were evaluated with CE-MRI/CT or CEUS to confirm the absence of tumoral foci at the resection margin. The patients in both groups underwent regular laboratory testing and medical imaging 1 month after treatment, then every 3 months during the first year, and every 6 months thereafter. Furthermore, bone scintigraphy, pelvic MRI, chest CT, or PET-CT was also performed in patients with extrahepatic metastasis according to their clinical symptoms or patients with unexplained a-fetoprotein (AFP) elevation. After LTP, if intrahepatic distant recurrence or extrahepatic metastasis was found during regular follow-up, surgical resection, ablation, radiation therapy, TACE, sorafenib, or liver transplantation were performed, depending on the tumor characteristics, liver function, or patients’ preferences.

### Comparison of therapeutic outcomes and definitions of terminology

LTP, overall survival (OS), disease-free survival (DFS), and cancer-specific survival (CSS) were analyzed. In the MWA group, the definition of LTP was enhancement at the arterial phase with washout at a delayed phase of CEUS, CECT, and CEMRI inside or abutting the ablation zone during follow-up. In the SR group, the LTP was defined as enhanced foci abutting the surgical margins. DFS was defined as the time during which no LTP, intrahepatic distant recurrence, extrahepatic metastasis, or death was detected after initial treatment. The calculation of the OS rate spanned from the date of the first treatment to either the date of death or the last visit to our outpatient clinic before November 30, 2019. The CSS rate was analyzed as HCC-specific mortality. Patient subgroup analysis was performed on the basis of characteristics including sex, age, and tumor size. Major complications were defined as clinical events leading to prolonged hospitalization or additional therapeutic interventions^27^.

### Statistical analysis

To decrease the overt bias of confounding factors between groups, we generated propensity scores with logistic regression and performed 1:1 patient matching according to each patient’s propensity score. The variables of age, sex, tumor size, tumor number, platelets, Child-Pugh score, end-stage liver disease (MELD), and albumin-bilirubin (ALBI) grade before ablation were included in the propensity score model with paired *t-*tests or the McNemar test for categorical variables and the Wilcoxon signed rank test for continuous variables. Cumulative incidence rates of OS, CSS, DFS, and LTP after PSM were also estimated with the Kaplan-Meier method, and differences between groups were compared with the log-rank test.

Baseline variables including patient characteristics, tumor characteristics, and post-treatment results between the MWA and SR groups were assessed with 2-tailed *t*-tests or the Mann-Whitney U test according to normality for continuous variables, and with χ^2^ test or Fisher exact test for categorical variables. The risks of CSS were calculated with Fine-and-Gray competing risk models, and death from non-HCC causes was considered a competing event. The Kaplan-Meier method was used to estimate the cumulative incidence rates of OS, CSS, DFS, and LTP, and the log-rank test was used to compare differences between groups.

Possible prognostic factors for OS, CSS, DFS, and LTP were estimated in univariate and multivariate analyses by using Cox proportional hazard models. Variables with *P* < 0.2 in univariable analyses were included in multivariable models. Subgroup analyses were performed to assess the homogeneity of the associations of OS, CSS, DFS, and LTP with the treatment modality in clinically relevant subgroups of patients in the matched cohort, on the basis of the Cox proportional hazard regression model. Stratification analysis was performed for important co-variates.

Major complications were carefully documented, and the rate differences between groups were compared with the McNemar test. Differences in post-treatment stay time in the 2 groups were calculated with 2-tailed *t*-test. Statistical analyses were performed with Empower (R) (www.empowerstats.com, X&Y Solutions, inc. Boston MA, USA) and R (http://www.R-project.org). Two-tailed probability values < 0.05 were considered statistically significant.

## Results

### Baseline characteristics

During the study period, 2,101 patients received SR or thermal ablation for HCC. Among them, 788 patients received MWA, and 1,025 received SR as a first-line treatment for HCC. A total of 729 patients were finally included in this study: 366 were in the MWA group and 363 in the SR group. The flow diagram of patient selection is shown in **Supplementary Figure S1**. The median follow-up was 78.6 months. Before PSM, patients in the MWA group were significantly older and had more liver tumors, more advanced liver disease (as evidenced by higher levels of the model for MELD, ALBI, and Child-Pugh score), whereas patients in the SR group had larger tumors. The PSM adjustment procedure of baseline characteristics generated 2 balanced groups of 253 patients each. The baseline characteristics of patients in the 2 groups are shown in **Table 1**, categorized before and after PSM.

**Table 1:** Baseline characteristics of study patients before and after propensity score analysis *n* (%)

Factor	Before propensity score matching			After propensity score matching		
	MWA group	SR group	*P*	MWA group	SR group	*P*
Age (years)	56.6 ± 10.1	54.0 ± 9.5	0.00	55.4 ± 10.1	55.2 ± 9.1	0.75
No. of men	288 (78.7)	288 (79.3)	0.83	201 (79.5)	203 (80.2)	0.83
Tumor size			0.00			0.18
≤ 3.0 cm	274 (74.9)	199 (54.8)		176 (69.6)	159 (62.9)	
> 3.0, ≤ 4.0 cm	61 (16.7)	122 (33.6)		48 (19.0)	65 (25.7)	
> 4.0, ≤ 5.0 cm	31 (8.5)	42 (11.6)		29 (11.4)	29 (11.4)	
Tumor number			0.00			0.39
1	301 (82.2)	335 (92.3)		222 (87.7)	228 (90.1)	
2~3	65 (17.8)	28 (7.7)		31 (12.3)	25 (9.8)	
Comorbidities			0.23			0.42
Diabetes	40 (10.9)	29 (8.0)		23 (9.1)	25 (9.9)	
Hypertension	63 (17.2)	56 (15.4)		43 (17.0)	40 (15.8)	
Hyperlipidemia	52 (14.2)	54 (14.9)		28 (11.1)	30 (11.9)	
Smoking status	201 (54.9)	198 (54.5)		135 (53.4)	122 (48.2)	
AFP level			0.00			0.93
< 200 ng/mL	322 (88.0)	274 (75.5)		227 (89.7)	223 (88.1)	
≥ 200 ng/mL	44 (12.0)	89 (24.5)		26 (10.3)	30 (11.9)	
Platelet			0.00			0.17
< 100/L	292 (79.8)	200 (55.1)		187 (73.9)	173 (68.4)	
≥ 100/L	74 (20.0)	163 (44.9)		66 (26.1)	80 (31.6)	
Hepatic virus			0.13			0.83
B	317 (86.6)	330 (90.9)		232 (91.7)	234 (92.5)	
C	30 (8.2)	21 (5.8)		21 (8.3)	19 (7.5)	
AST (U/L)	35.9 ± 18.3	35.5 ± 21.8	0.34	35.2 ± 17.3	34.9 ± 17.9	0.67
Child-Pugh score			0.00			0.22
A	339 (92.6)	357 (98.3)		247 (97.6)	242 (95.6)	
B	27 (7.4)	6 (1.7)		6 (2.4)	11 (4.4)	
MELD			0.00			0.38
≤ 8	109 (29.8)	163 (44.9)		107 (42.3)	93 (36.8)	
≤ 10	195 (53.3)	175 (48.2)		123 (48.6)	131 (51.8)	
≤ 20	62 (16.9)	25 (6.9)		23 (9.1)	29 (11.4)	
ALBI			0.00			0.21
≤ −2.6	162 (44.3)	250 (68.9)		126 (49.8)	115 (45.5)	
≤ −1.39	183 (50.0)	110 (30.3)		114 (45.1)	125 (49.4)	
≤ −0.01	21 (5.7)	3 (0.8)		13 (5.1)	13 (5.1)	

Data are means ± standard deviations or medians with interquartile ranges in parentheses for continuous variables and are numbers of patients with percentages in parentheses for categorical variables. AFP, alpha fetoprotein; AST, aspartate aminotransferase; MELD, Model for End-stage Liver Disease; ALBI, albumin-bilirubin grade; MWA, microwave ablation; SR, surgical resection.

After PSM, all 253 patients in the SR group had undergone R0 resection and histopathologic analysis, which confirmed the complete resection of tumor cells. A total of 239 patients achieved complete coagulation after 1 session of MWA, and 14 patients achieved complete coagulation after 2 sessions. HCC was pathologically demonstrated for 128 (50.6%) patients according to percutaneous biopsy during or before ablation, or according to non-invasive criteria^19^ for the remaining 125 (49.4%) patients in the MWA group.

### Comparison of therapeutic outcomes after PSM

#### OS

During the follow-up period of 72.4 months, 86 of 253 (34.0%) patients in the MWA group and 78 of 253 (30.8%) patients in the SR group died. For ≤ 5.0 cm tumors, the 1-, 3-, and 5-year OS rates were estimated to be 98.4%, 87.3%, and 76.0%, respectively, in the SR group, and 96.4%, 83.0%, and 72.7%, respectively, in the MWA group (**Supplementary Figure S2A**). MWA was associated with poorer OS in the matched cohort, but the difference was not significant [hazard ratio (HR), 1.32; 95% confidence interval (CI), 0.97, 1.79, *P* = 0.08] (**Supplementary Table S1**). MWA and SR had similar risks of death for ≤ 3.0 cm tumors (HR, 1.21; 95% CI, 0.79, 1.83; *P* = 0.38) and 3.1–4.0 cm (HR, 1.12; 95% CI, 0.65, 1.92; *P* = 0.69) (**Table 2, Supplementary Figure S2B and S2C**). However, for 4.1–5.0 cm tumors, MWA was associated with a higher risk of death than SR (HR, 3.35; 95% CI, 1.31, 8.58; *P* = 0.01) (**Table 2, Supplementary Figure S2D**).

**Table 2:** Risk of main overall outcomes in the propensity score matched Cohort of tumors ≤ 3.0 cm, 3.1–4.0 cm and 4.1–5.0 cm

Outcomes	PE	≤ 3.0 cm HR (95% CI)	*P*	PE	3.1–4.0 cm HR (95% CI)	*P*	PE	4.1–5.0 cm HR (95% CI)	*P*
OS									
SR	42	Reference	0.38	30	Reference	0.69	6	Reference	0.01
MWA	47	1.21 (0.79, 1.83)		23	1.12 (0.65, 1.92)		16	3.35 (1.31, 8.58)	
CSS									
SR	31	Reference	0.82	19	Reference	0.98	3	Reference	0.01
MWA	28	0.94 (0.56, 1.58)		13	1.01 (0.50, 2.04)		12	5.01 (1.41, 17.76)	
DFS									
SR	85	Reference	0.59	33	Reference	0.93	11	Reference	0.04
MWA	81	0.92 (0.68, 1.25)		27	1.02 (0.61, 1.70)		17	2.18 (1.01, 4.68)	
LTP									
SR	1	Reference	0.95	1	Reference	0.12	1	Reference	0.21
MWA	1	0.92 (0.06, 14.65)		4	5.57 (0.62, 49.87)		4	4.10 (0.46, 36.66)	

PE, patients with event; HR, hazard rate; OS, overall survival; CSS, cancer specific survival; DFS, disease free survival; LTP, local tumor progression; MWA, microwave ablation; SR, surgical resection.

#### CSS

**Figure 1:**
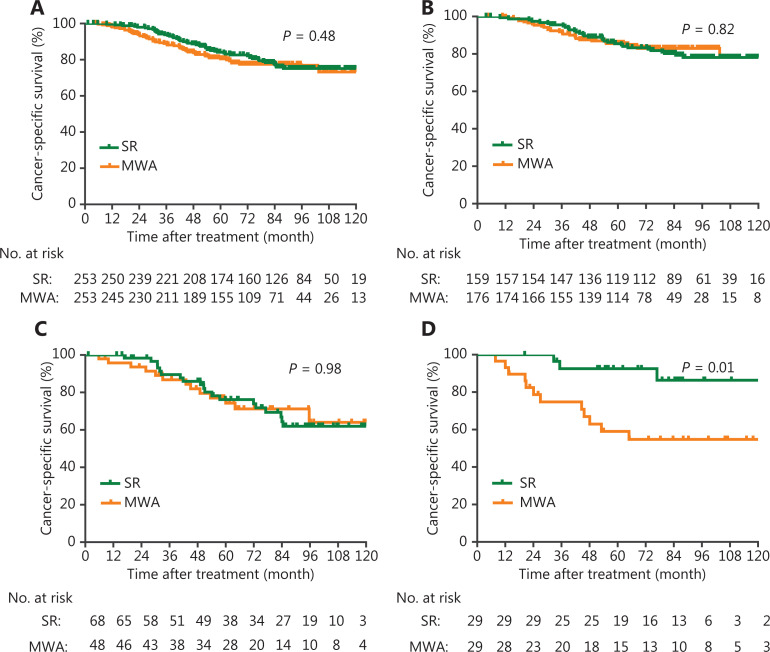
Kaplan-Meier CSS curves of patients in a propensity score matched cohort. (A) tumors ≤ 5.0 cm; (B) tumors ≤ 3.0 cm; (C) tumors 3.1–4.0 cm; (D) tumors 4.1–5.0 cm. Dashed line = 50% survival.

For ≤ 5.0 cm tumors, MWA was associated with an equivalent CSS rate to that of SR in the matched cohort (HR, 1.19; 95% CI, 0.81, 1.75, *P* = 0.37) (**Supplementary Table S1, Figure 1A**). No significant differences were found in CSS between groups in both ≤ 3.0 cm tumors (HR, 0.94; 95% CI, 0.56, 1.58; *P* = 0.82) and 3.1–4.0 cm tumors (HR, 1.01; 95% CI, 0.50, 2.04; *P* = 0.98) (**Table 2, Figure 1B and C**). However, for 4.1–5.0 cm tumors, MWA was associated with a higher risk of cancer-specific death than SR (HR, 5.01; 95% CI, 1.41, 17.76; *P* = 0.01) (**Table 2, Figure 1D**).

#### DFS

MWA was associated with similar 5-year DFS among patients with ≤ 5.0 cm tumors in the matched cohort (HR, 1.03; 95% CI, 0.81, 1.32; *P* = 0.80) (**Supplementary Table S1**). The 1-, 3-, and 5-year DFS rates were estimated to be 80.54%, 58.90%, and 52.63%, respectively, in the MWA group, and 82.78%, 63.81%, and 50.93%, respectively, in the SR group (**Supplementary Figure S3**). In subgroup analysis, no statistical significance was found in the DFS rates of tumors ≤ 3.0 cm (HR, 0.92; 95% CI, 0.68, 1.25; *P* = 0.59) and 3.1–4.0 cm (HR, 1.02; 95% CI, 0.61, 1.70; *P* = 0.93), but MWA was found to be associated with a higher risk of tumor progression than SR for 4.1–5.0 cm tumors (HR, 2.18; 95% CI, 1.01, 4.68; *P* = 0.04) (**Table 2, Supplementary Figure S3**).

#### LTP

A total of 9 of 253 (3.56%) patients in the MWA group and 3 of 253 (1.19%) patients in the SR group showed LTP (**Supplementary Table S2**). No significant difference was found between groups (HR, 3.75; 95% CI, 0.75, 18.68; *P* = 0.11) (**Supplementary Table S1, Supplementary Figure S4**). In subgroup analysis, no statistically significant differences were found in the LTP rates for tumors smaller than 3.0 cm, 3.1–4.0 cm, and 4.1–5.0 cm (all *P* > 0.05) (**Supplementary Table S2**).

#### Subgroup analysis of anatomic and non-anatomic SR

In subgroup analysis of SR, anatomic resection showed better 5-year OS (HR, 0.28; 95%CI, 0.08, 0.97; *P* = 0.04) and CSS (HR, 0.13; 95%CI, 0.02, 0.98; *P* = 0.04) than those of MWA in 4.1–5.0 cm tumors in the matched cohort, whereas non-anatomic resection showed similar OS, CSS, and DFS to those of MWA in all size subgroups (**Supplementary Table S3**). No significant differences were found in the risk of local tumor progression among the 3 groups in all tumor size categories (**Supplementary Table S2**).

### Uni- and multivariate analyses for OS, CSS, DFS, and LTP

The predictors of OS, CSS, DFS, and LTP in uni- and multivariate analyses are shown in **Supplementary Tables S4–S6 and Table 3**. Multivariate analysis showed that the significant prognostic factors were tumor size (HR, 1.46; 95% CI: 1.18, 1.80; *P* < 0.01) and Child-Pugh score (HR, 3.76; 95% CI: 2.04, 6.93; *P* < 0.01) for OS; tumor size (HR, 1.42; 95% CI: 1.10, 1.85; *P* < 0.01) for CSS; tumor number (HR, 2.05; 95% CI: 1.36, 2.80; *P* < 0.01) and Child-Pugh score (HR, 2.18; 95% CI: 1.16, 4.08; *P* = 0.02) for DFS; and tumor size (HR, 3.62; 95% CI: 1.73, 7.57; *P* < 0.01) for LTP.

**Table 3:** Univariate and multivariate analyses of CSS

Factors	Univariate analysis			Multivariate analysis		
	HR	95% CI	*P*	HR	95% CI	*P*
Age	1.02	0.99, 1.04	0.14			
Gender	0.99	0.62, 1.60	0.97			
Tumor size	1.39	1.08, 1.78	< 0.01	1.42	1.10, 1.85	0.01
Number	1.09	0.60, 1.99	0.78			
Virus	1.17	0.59, 2.32	0.65			
AFP	0.88	0.54, 1.45	0.62			
AST	1.18	0.80, 1.75	0.40			
Child-Pugh score	1.48	0.47, 4.69	0.50			
MELD	0.85	0.62, 1.16	0.30			
ALBI	1.13	0.81, 1.58	0.47			
Treatment	1.15	0.78, 1.69	0.48			

AFP, alpha fetoprotein; AST, aspartate aminotransferase; MELD, Model for End-stage Liver Disease; ALBI, albumin-bilirubin grade.

### Stratification analysis by multivariable Cox regression for OS, CSS, and DFS

**Figure 2:**
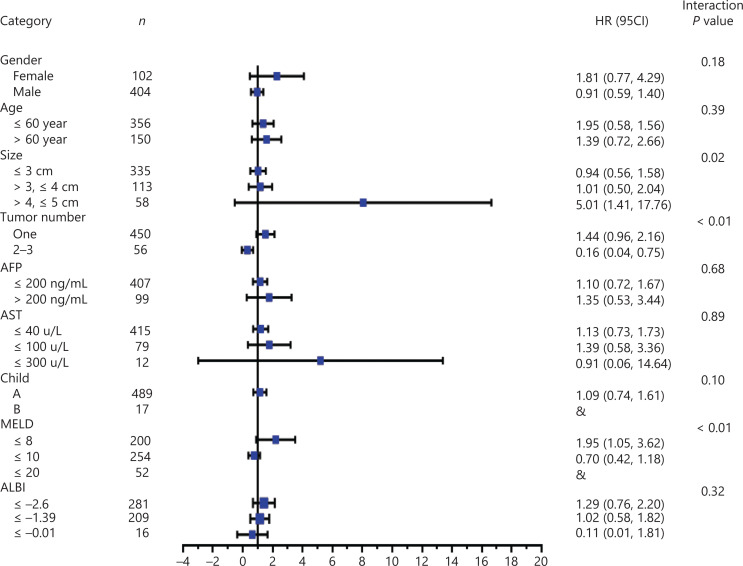
Stratification analysis by multivariable Cox regression results of cancer-specific survival. CI, confidence interval. AFP, alpha fetoprotein; AST, aspartate aminotransferase; MELD, Model for End-stage Liver Disease; ALBI, albumin-bilirubin grade.

Multivariable Cox regression analysis showed the therapeutic effect modification by clinical characteristics and presentations. The association of MWA with the composite of all-cause death was stronger among patients with a solitary 4.1–5.0 cm tumor (HR: 1.45; 95% CI: 1.04, 2.02; *Pint* = 0.01) (**Supplementary Figure S5**). The survival time associated with HCC was 4.01 times longer when SR was used as a curative therapy rather than MWA for 4.1–5.0 cm HCC (HR: 5.01; 95% CI: 1.41, 17.76; *Pint* = 0.02), and the HCC specific death rates were lower when MWA was used as the curative therapy (HR: 0.16; 95% CI: 0.04, 0.75; *Pint* < 0.01) (**Figure 2**). No evidence was found regarding the therapeutic effects on the modification of disease progression. (**Supplementary Figure S6**).

### Intraoperative and postoperative outcomes

The rates of major complications did not differ between the MWA and SR groups either before (3.01% *vs.* 4.96%, *P* = 0.19) or after PSM (2.77% *vs.* 4.34%, *P* = 0.41), according to the Society of Interventional Radiology classification (**Table 4**). The complications were all directly associated with the treatment procedures. No treatment related deaths were detected in either group, and post-hepatectomy liver failure was not observed in the SR group. In addition, the length of post-treatment stay was significantly longer in the SR group than the MWA group.

**Table 4:** Complications after MWA and SR

Complications	All data			Matched data		
	MWA group	SR group	*P*	MWA group	SR group	*P*
Treatment-related death	0	0	–	0	0	–
Post-treatment stay (d)	7 (3–21)	10 (4–29)	0.00	6 (4–19)	10 (4–28)	0.00
Estimated blood loss (mL)	21.3	309.1	0.00	20.3	303.2	0.00
Major complications						
Pleural effusion	4	3		3	2	
Tumor seeding	2	1		1	0	
Hepatic abscess	1	2		0	1	
Bile injury	1	2		0	1	
Bleeding	1	5		1	4	
Ascites	2	5		2	3	
Total	11	18	0.19	7	11	0.41

MWA, microwave ablation; SR, surgical resection.

## Discussion

To our knowledge, our study has the largest patient number and longest follow-up time among published comparison studies of MWA and SR. All patients received MWA or SR treatment for naïve HCC. No differences were found regarding the OS, CSS, DFS, and LTP in ≤ 4.0 cm tumors after MWA or SR. SR appeared to be superior in patients with 4.1–5.0 cm HCC, in terms of OS (HR, 3.35; 95% CI, 1.31, 8.58; *P* = 0.01), CSS (HR, 5.01; 95% CI, 1.41, 17.76; *P* = 0.01), and DFS (HR, 2.18; 95% CI, 1.01, 4.68; *P* = 0.04). Patients with MWA experienced less blood loss and shorter hospital stays than those with SR. The complication rate was relatively higher with SR (2.77% *vs.* 4.34%, *P* = 0.41), although the difference was not significant.

SR and thermal ablation are 2 treatment modalities for patients with HCC who are not eligible or waiting for liver transplantation^1^. Thermal ablation is a less invasive procedure associated with a lower liver disease decompensation. Recent studies comparing the therapeutic outcomes of thermal ablation and SR have focused primarily on RFA and SR. Randomized controlled trials and large-scale retrospective studies have fully demonstrated the equivalent oncological results for RFA and SR for HCCs smaller than 3.0 cm^28–32^; however, for intermediate-sized tumors of 3.0–5.0 cm, controversial results exist because of the limitations of the lower efficacy of RFA and the different types of RFA equipment used^33–36^.

MWA is a promising new thermal technique whose theoretical advantages over RFA, including its high thermal efficiency and lower sensitivity to heat sink effects, have been validated in numerous studies. Two randomized controlled trials have shown that the OS with MWA is comparable to that with RFA for 3.0–5.0 cm HCC, whereas MWA requires fewer sessions than RF^13,37^, in agreement with its potentially larger ablation zone and more comprehensive tumor coverage of MWA.

Data on the direct comparison of MWA and SR are limited and have led to different conclusions regarding the treatment of HCC (**Supplementary Table S7**). In ≤ 3.0 cm HCC, no significant differences have been found in OS rates between MWA and SR, although SR has been reported to be associated with higher DFS rates than MWA^17,38,39^. One study has found similar DFS rates for ≤ 3.0 cm HCC^18^. Recent studies on 3.0–5.0 cm HCC have been controversial, and no detailed subgroup analyses had been available^17,18^. In addition, those results focused on OS and DFS rates, but other oncological results, such as CSS and LTP rates between groups, were unavailable.

In this large-scale retrospective study, according to tumor size, we summarized the oncologic and functional results with at least 5-year follow-up for patients with early-stage HCC who received treatment naïve MWA or SR. To control the bias and confounding of baseline data between the MWA and SR groups, we balanced demographics, tumor characteristics and liver function between groups before analysis. We found that not only the ≤ 3.0 cm HCC group but also the 3.1–4.0 cm HCC group showed comparable long-term therapeutic efficacy between MWA and SR in terms of OS, CSS, and DFS. Poorer OS, CSS, and DFS were observed with MWA for 4.1– 5.0 cm tumors. Those results may be associated with the significantly increased tumor volume hindering complete ablation. Although doctors in our center are very experienced in image-guided MWA, and we have been ablating 3–5 cm tumors since 1995^16^, MWA still tends to be inferior to SR for treating larger tumors, because SR provides a substantial safety margin. In addition, the rate of microvascular invasion increases relative to tumor size^33^, and thus distant metastases are more likely to occur, thereby negatively influencing DFS and CSS. The LTP rate was also higher with MWA than SR for 4.1–5.0 cm tumors, although the difference was not significant. The sample in the subgroup with a tumor diameter > 3.0 cm was small, and few LTP cases were found. Further studies with more cases are therefore needed to validate this conclusion. Our previous study analysis of 2,529 tumors concluded that LTP does not affect patients’ survival prognosis^40^. This finding might explain why the LTP rate showed no statistical difference, but the CSS did show a significant difference, given that intrahepatic recurrence and extrahepatic recurrence (DFS) play important roles in CSS and OS.

In contrast with previous studies, we clarified tumor size according to 3 categories in the present study. We further subdivided tumors with sizes between 3.0–5.0 cm, because an increase in 1 cm in tumor diameter significantly increases the energy needed to ablate the whole tumor in 3-dimensional (3D) space. The volumes are estimated to be 14.1 mL, 33.5 mL, and 65.4 mL in tumors with a maximum diameter of 3.0 cm, 4.0 cm, and 5.0 cm, respectively. Every 1 cm increase in maximum tumor diameter results in an approximately 2-fold increase in tumor volume. For tumors larger than 3.0 cm, a combination of at least 4 ablation zones typically required to overlap the entire tumor in 3D space for MWA; this aspect remains a major challenge requiring further researches^41^. With the development of 3D visualization plans and modern MWA equipment in recent years, great progress has been made in MWA in local tumor control^42,43^. Future data with new techniques for large tumors are expected to highlight the power of MWA.

Our study has several limitations. First, our retrospective study design may have led to selection bias. There is a possibility of uncontrolled confounding factors between the MWA and SR group, although we attempted to simulate randomization by PSM. Second, less than half the patients were diagnosed with biopsy-validated HCCs in the MWA group. Pathological results were not analyzed for relationships with prognostic outcomes. Third, after subgroup analysis, the patient number was small for 4.1–5.0 cm HCC. Further studies with larger patient numbers and more strict analysis are needed to illustrate the oncologic results of MWA and SR in 4.1–5.0 cm HCC. Fourth, this was a single-center study. Therefore, our results might not be reproducible in other settings. Our findings may provide additional clinical evidence that compares MWA with SR through PSM in a large retrospective cohort with long-term follow-up. In addition, we could not exclude the potential of selection bias, owing to the limited number of patients with 4.1–5.0 cm HCC after subgroup group analysis.

In conclusion, microwave ablation serve as a minimally invasive alternative to surgical resection for the treatment of HCC smaller than 4.0 cm, providing the advantages of low invasiveness and faster recovery.
