# Supplementary material for: Microwave ablation vs. surgical resection for treatment naïve hepatocellular carcinoma within the Milan criteria: a follow-up of at least 5 years
Source: Cancer Biol Med. 2021 Sep 30;19(7):1078–88. doi: 10.20892/j.issn.2095-3941.2020.0625 (PMC9334764; doi:10.20892/j.issn.2095-3941.2020.0625)
Supplement: Supplementary file 1 [file cbm-19-1078-s001.pdf]

Supplementary materials

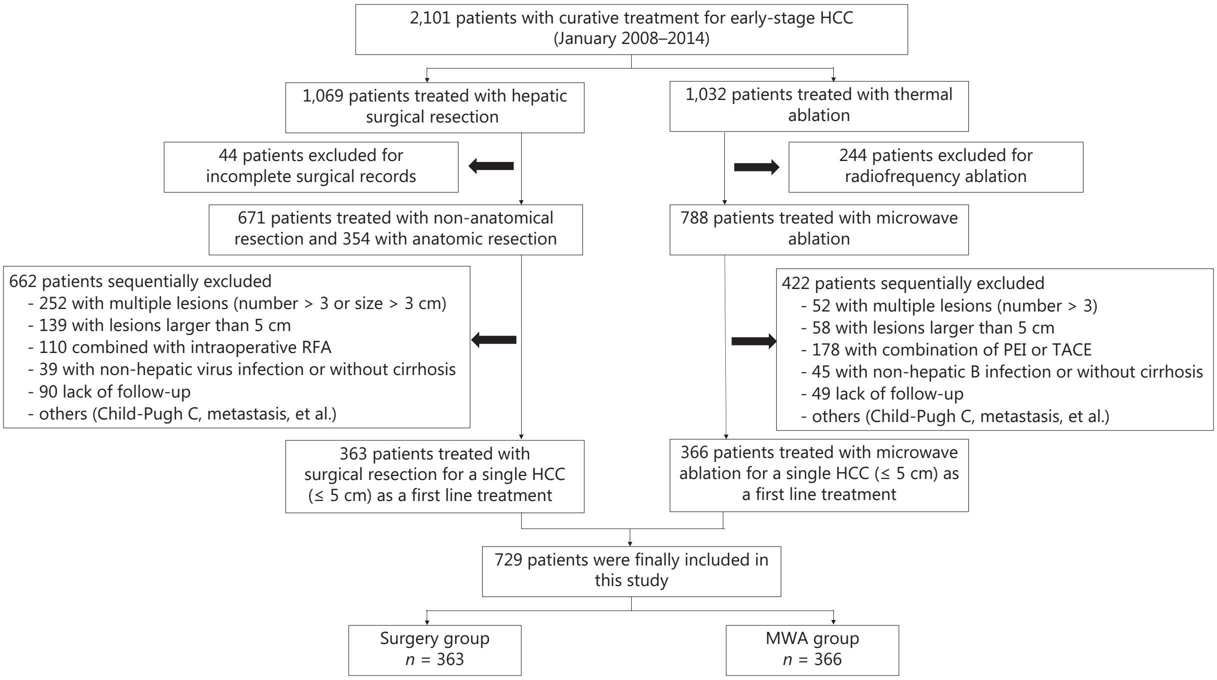

Figure S1 Flow diagram of patient selection in this study. PEI, percutaneous ethanol injection; TACE, transcatheter arterial chemoembolization. MWA, microwave ablation.

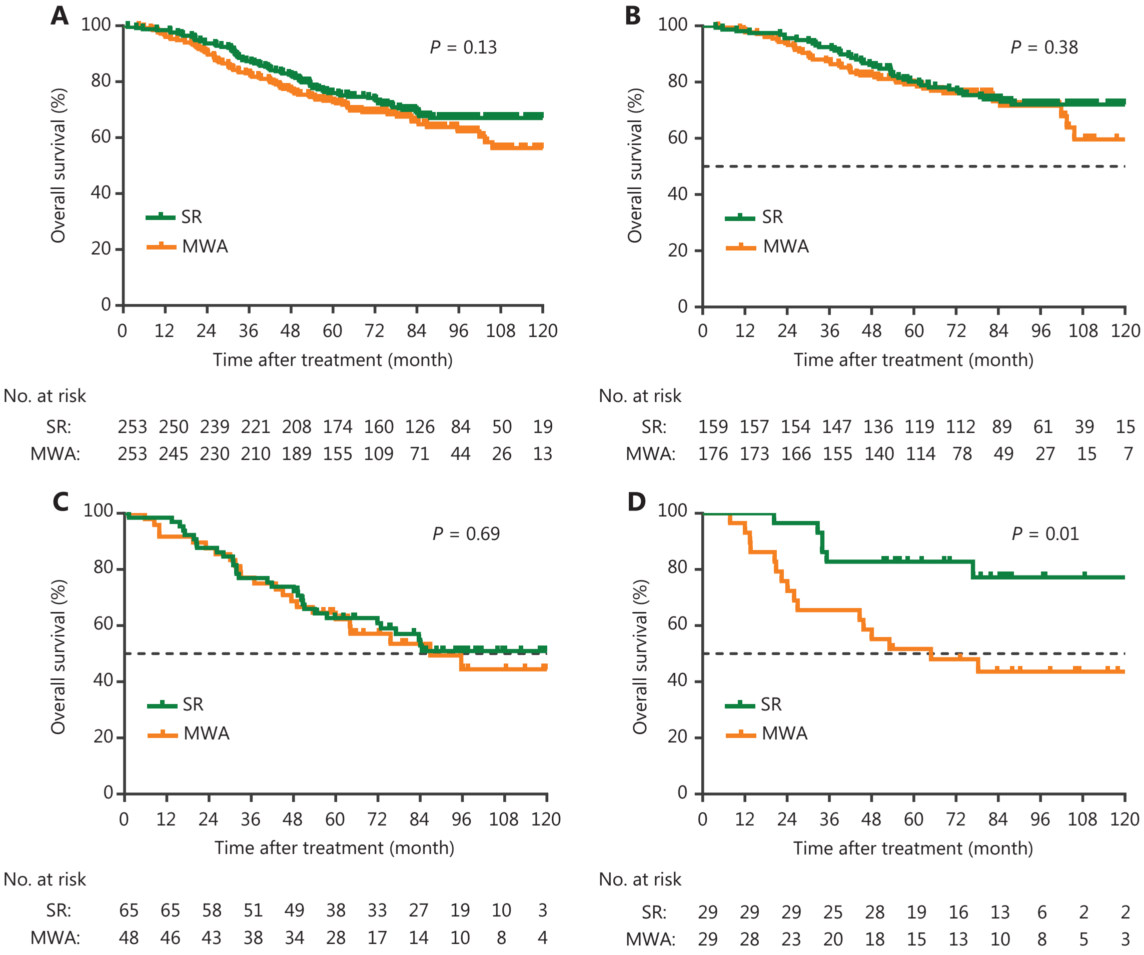

Figure S2 Kaplan-Meier OS curves of patients in the propensity score matched cohort. (A) tumors ≤ 5.0 cm; (B) tumors ≤ 3.0 cm; (C) tumors 3.1–4.0 cm; (D) tumors 4.1–5.0 cm. Dashed line = 50% survival. SR, surgical resection; MWA, microwave ablation.

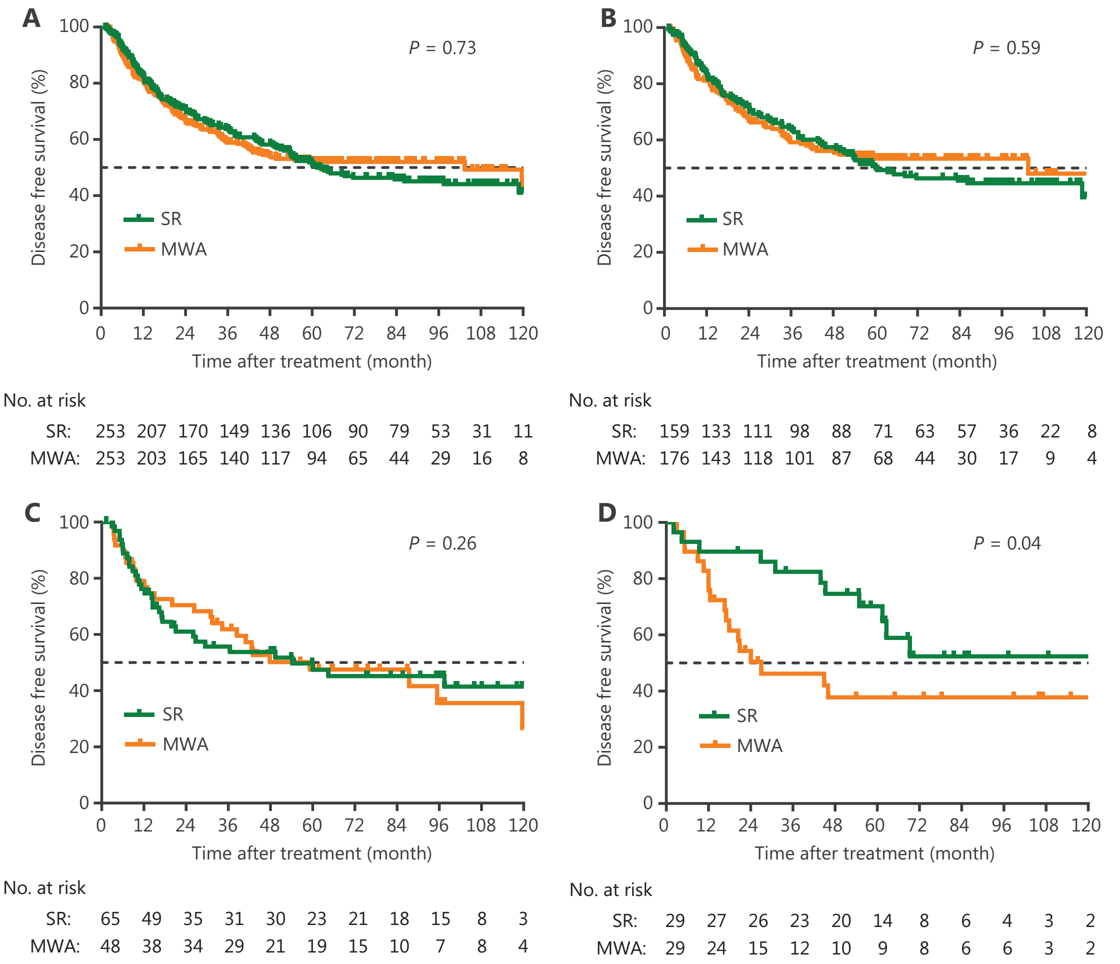

Figure S3 Kaplan-Meier DFS curves of patients in the propensity score matched cohort. (A) tumors ≤ 5.0 cm; (B) tumors ≤ 3.0 cm; (C) tumors 3.1~4.0 cm; (D) tumors 4.1–5.0 cm. Dashed line = 50% survival. SR, surgical resection; MWA, microwave ablation.

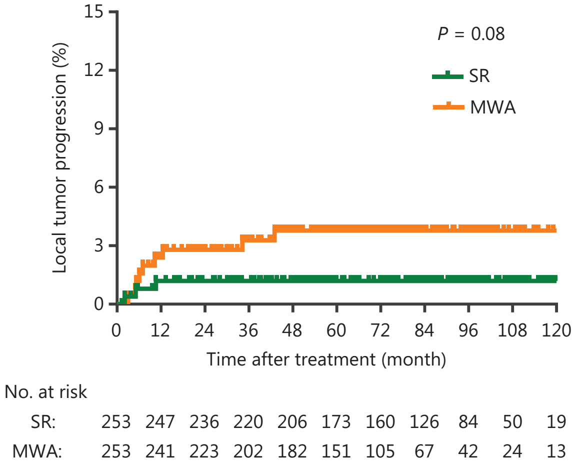

Figure S4 Kaplan-Meier survival curves of cumulative LTP for patients who underwent microwave ablation (MWA) or surgical resection (SR) in the propensity score matched cohort. SR, surgical resection; MWA, microwave ablation.

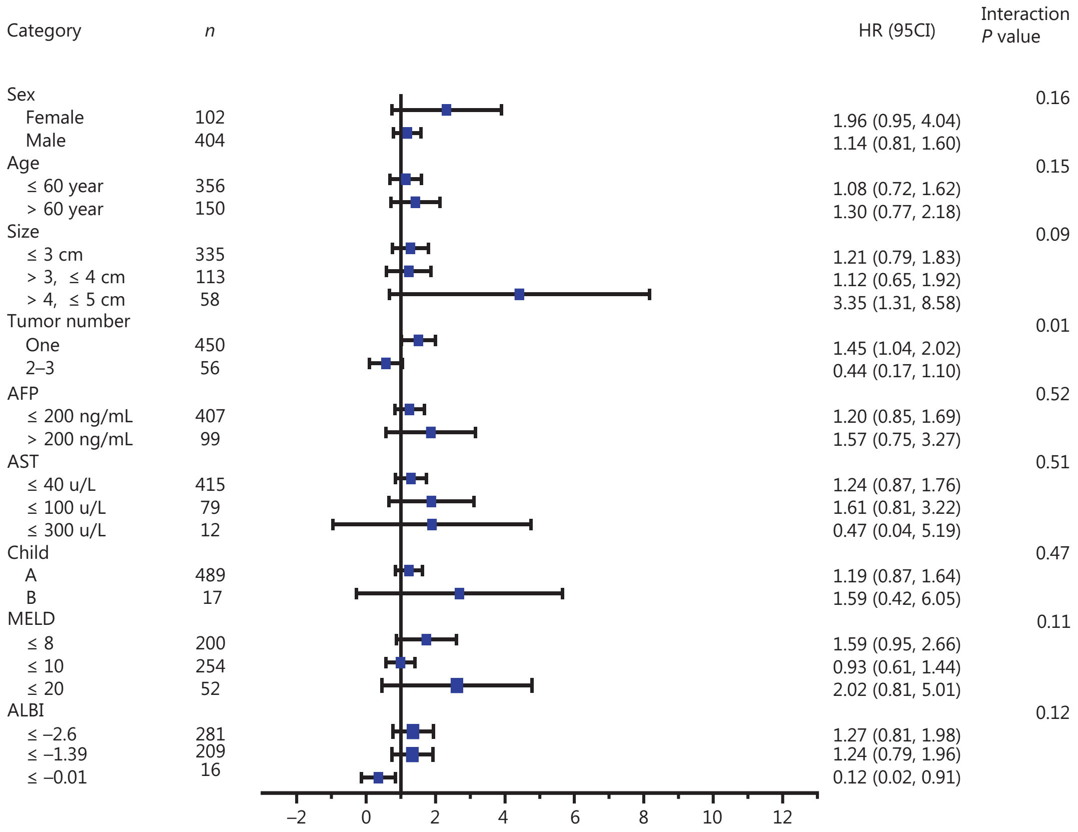

Figure S5 Stratification analysis by multivariable Cox regression results of overall survival. CI, confidence interval. AFP, alpha fetoprotein; AST, aspartate aminotransferase; MELD, Model for End-stage Liver Disease; ALBI, albumin-bilirubin grade.

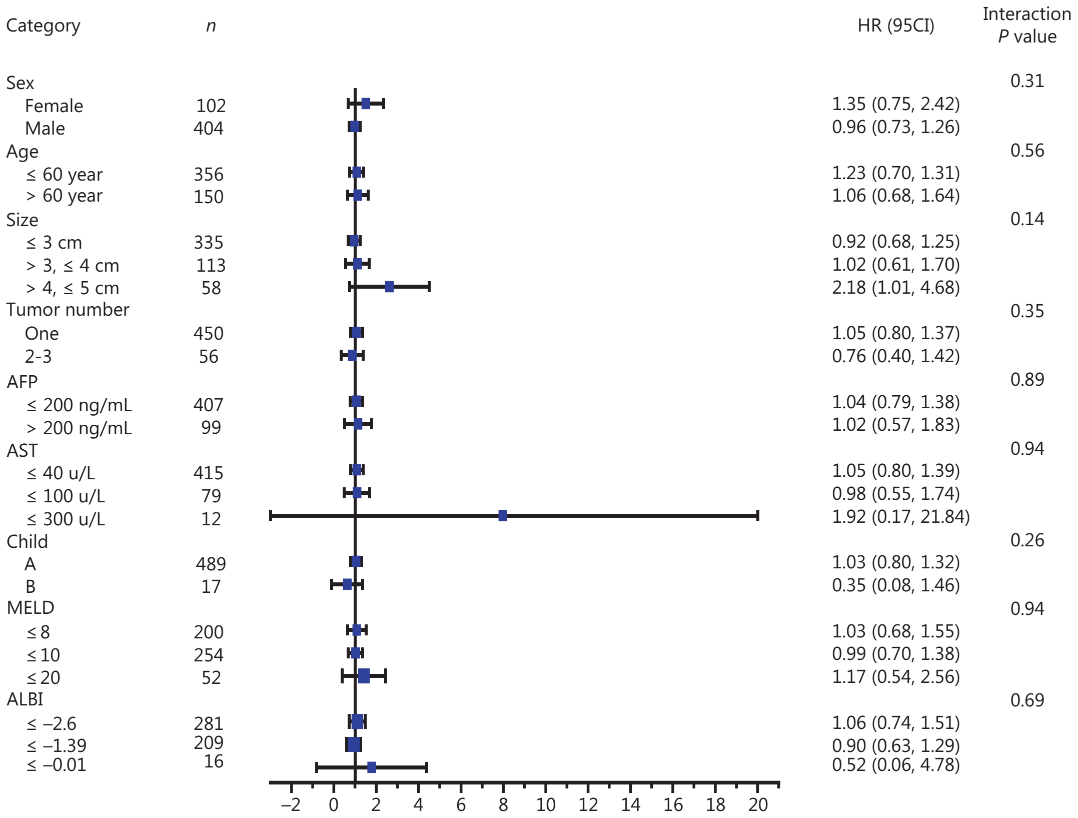

Figure S6 Stratification analysis by multivariable Cox regression results of disease-free survival. CI, confidence interval. AFP, alpha fetoprotein; AST, aspartate aminotransferase; MELD, Model for End-stage Liver Disease; ALBI, albumin-bilirubin grade.

Table S1 Risk of main outcomes in the propensity score matched cohort of all tumors

Outcomes	No. of patients with event	ER (%/yr)	HR (95% CI)	*P*
OS				
SR	78	12.00	Reference	0.08
MWA	86	15.47	1.32 (0.97, 1.79)	
CSS				
SR	53	8.15	Reference	0.37
MWA	53	9.53		
DFS			1.19 (0.81, 1.75)	
SR	129	19.85	Reference	0.80
MWA	125	22.48	1.03 (0.81, 1.32)	
LTP				
SR	3	0.46	Reference	0.11
MWA	9	1.62	3.75 (0.75, 18.68)	

ER, event rate; HR, hazard rate; OS, overall survival; CSS, cancer specific survival; DFS, disease free survival; LTP, local tumor progression; MWA, microwave ablation; SR, surgical resection.

Table S2 Risk of local tumor progression in subgroup analysis

Local tumor progression	MWA (*n* = 253)	Surgery (*n* = 253)	Subgroup analysis of surgery		*P* (MWA *vs*. surgery)	*P* (subgroup analysis of surgery)
			Anatomic (*n* = 95)	Non-anatomic (*n* = 158)		
≤ 3.0 cm	1/175	1/158	0/52	1/107	0.94	0.77
	(0.57)	(0.63)	(0.00)	(0.93)		
> 3.0, ≤ 4.0 cm	4/44	1/64	0/27	1/38	0.08	0.20
	(8.33)	(1.54)	(0.00)	(2.63)		
> 4.0, ≤ 5.0 cm	4/28	1/29	0/16	1/13	0.16	0.29
	(15.50)	(3.45)	(0.00)	(7.69)		
Total	9/253	3/253	0/95	3/158	0.08	0.14
	(3.56)	(1.19)	(0.00)	(1.90)		

Data are numbers of patients with percentages in parentheses for categorical variables. MWA, microwave ablation; SR, surgical resection.

Table S3 Risk of overall survival, cancer specific survival and disease free survival in subgroup analysis

Outcomes	PE	≤ 3.0 cm HR (95% CI)	*P*	PE	3.1–4.0 cm HR (95% CI)	*P*	PE	4.1–5.0 cm HR (95% CI)	*P*
OS									
MWA	47	Reference		23	Reference		16	Reference	
Anatomic	11	0.67 (0.35, 1.30)	0.24	11	0.89 (0.43, 1.83)	0.75	3	0.28 (0.08, 0.97)	0.04
Non-anatomic	31	0.90 (0.57, 1.43)	0.67	19	0.90 (0.49, 1.65)	0.73	3	0.31 (0.09, 1.08)	0.06
CSS									
MWA	28	Reference		13	Reference		12	Reference	
Anatomic	9	0.94 (0.45, 2.00)	0.88	7	1.01 (0.40, 2.54)	0.98	1	0.13 (0.02, 0.98)	0.04
Non-anatomic	22	1.12 (0.64, 1.96)	0.70	12	0.98 (0.45, 2.16)	0.96	2	0.28 (0.06, 1.25)	0.09
DFS									
MWA	81	Reference		27	Reference		17	Reference	
Anatomic	28	1.14 (0.74, 1.75)	0.55	11	0.79 (0.39, 1.60)	0.51	5	0.39 (0.14, 0.98)	0.06
Non-anatomic	57	1.06 (0.76, 1.49)	0.72	22	1.11 (0.63, 1.95)	0.72	6	0.54 (0.21, 1.37)	0.19

PE, Patients with event; HR, hazard rate; OS, overall survival; CSS, cancer-specific survival; DFS, disease free survival; MWA, microwave ablation; SR, surgical resection.

Table S4 Univariate and multivariate analyses of OS

Factors	Univariate analysis			Multivariate analysis		
	HR	95% CI	*P*	HR	95% CI	*P*
Age	1.01	0.99, 1.02	0.22			
Gender	1.01	0.86, 1.48	0.97			
Tumor size	1.41	1.15, 1.72	< 0.01	1.46	1.18, 1.80	< 0.01
Number	1.25	0.79, 1.98	0.33			
Virus	1.36	0.81, 2.28	0.24			
AFP	0.90	0.61, 1.34	0.62			
AST	1.24	0.91, 1.24	0.17			
Child-Pugh score	3.97	2.19, 7.18	< 0.01	3.76	2.04, 6.93	< 0.01
MELD	1.19	0.94, 1.52	016			
ALBI	1.29	0.99, 1.68	0.05	1.10	0.82, 1.60	0.53
Treatment	1.26	0.93, 1.72	0.13			

AFP, alpha fetoprotein; AST, aspartate aminotransferase; MELD, Model for End-stage Liver Disease; ALBI, albumin-bilirubin grade.

Table S5 Univariate and multivariate analyses of DFS

Factors	Univariate analysis			Multivariate analysis		
	HR	95% CI	*P*	HR	95% CI	*P*
Age	1.03	0.80, 1.31	0.83			
Gender	1.18	0.86, 1.63	0.3			
Tumor size	1.04	0.87, 1.23	0.69			
Number	1.82	1.29, 2.56	< 0.01	2.05	1.36, 2.80	< 0.01
Virus	0.98	0.62, 1.57	0.94			
AFP	1.07	0.79, 1.46	0.67			
AST	1.12	0.87, 1.45	0.36			
Child-Pugh score	2.13	1.16, 3.91	0.01	2.18	1.16, 4.08	0.02
MELD	1.11	0.91, 1.35	0.27			
ALBI	1.28	1.04, 1.57	0.02	1.23	0.97, 1.56	0.08
Treatment	1.03	0.80, 1.31	0.83			

AFP, alpha fetoprotein; AST, aspartate aminotransferase; MELD, Model for End-stage Liver Disease; ALBI, albumin-bilirubin grade.

Table S6 Univariate and multivariate analyses of LTP

Factors	Univariate analysis			Multivariate analysis		
	HR	95% CI	*P*	HR	95% CI	*P*
Age	1.05	0.98, 1.13	0.14			
Gender	0.50	0.15, 1.67	0.26			
Tumor size	3.57	1.79, 7.15	< 0.01	3.62	1.73, 7.57	< 0.01
Number	0.74	0.10, 5.70	0.74			
Virus	6.05	1.82, 20.11	< 0.01	3.15	0.73, 13.58	0.12
AFP	1.38	0.37, 5.09	0.63			
AST	0.85	0.22, 3.31	0.81			
Child-Pugh score	1.33	0.18, 10.0	0.78			
MELD	0.92	0.37, 2.24	0.85			
ALBI	1.80	0.71, 4.58	0.22			
Treatment	3.05	0.83, 11.28	0.09	2.99	0.73, 12.33	0.13

AFP, alpha fetoprotein; AST, aspartate aminotransferase; MELD, Model for End-stage Liver Disease; ALBI, albumin-bilirubin grade.

Table S7 Comparing studies of SR and MWA in HCC treatment

Author	Year and region	Study design	Number of patients*	Diameter (cm)	Median follow-up (months)*	OS (3/5-year)	CSS (3/5-year)	DFS (3/5-year)	LTP (%)	Complications rate (%)
Zhang et al.[38]	2016 China	Retrospective cohort study	122/68	≤ 3.0 cm	39.5/37	No difference	NA	SR better	NA	MWA lower
Li et al.[39]	2017 China	Retrospective cohort study	220/60	≤ 3.0 cm	NA	No difference	NA	SR better	NA	MWA lower
Wang et al.[16]	2008 China	Retrospective cohort study	80/114	≤ 5.0 cm (without subgroup of size)	NA	No difference	NA	No difference	NA	No difference
Liu et al.[17]	2019 China	Retrospective cohort study	212/116	≤ 5.0 cm	37.8/36.8	≤ 3.0 cm: no difference; 3.1–5 cm: SR better	NA	SR better	NA	NA
Shi et al.[18]	2014 China	Retrospective cohort study	107/117	≤ 5.0 cm	NA	No difference	NA	≤ 3 cm: no difference; 3.1–5.0 cm: SR better	NA	NA

*SR/MWA. OS, overall survival; CSS, cancer specific survival; DFS, disease free survival; LTP, local tumor progression; MWA, microwave ablation; SR, surgical resection.
